# Supplementary material for: Participatory Systems Modelling for Youth Mental Health: An Evaluation Study Applying a Comprehensive Multi-Scale Framework
Source: Int J Environ Res Public Health. 2022 Mar 28;19(7):4015. doi: 10.3390/ijerph19074015 (PMC8998357; doi:10.3390/ijerph19074015)
Supplement: Supplementary file 1 [file ijerph-19-04015-s001.zip › Lee GY_PSM evaluation plan YMH Program_SUPPLEMENTARY DOC 1.pdf]

***Right care, first time, where you live:* Developing a *'what if' tool* to improve youth mental health in [*name of participating site*]**

PRE-WORKSHOP 1 SURVEY

Thank you for taking the time to fill out this 15-minute survey. You have been invited to participate in this research because we believe that your experiences are important, and we want your voice to be heard. You will receive a $20 voucher for a retail provider of your choice upon completion of this survey.

Our research aims to improve mental health care for young people (e.g. easier to find) in the [name of participating site] region. As part of this research, you are invited to three (3) co-design workshops where you will interact with many different people from various backgrounds to discuss the challenges and solutions for youth mental health care. Co-design means that you will be considered an equal to everyone in the workshops – your experiences and opinions will be listened to and everyone will be treated with respect.

*During the co-design workshops, we will work together to develop a **'what if' tool** using a research method called systems modelling.*

*Systems modelling allows people to understand '**what if'** scenarios to help make better decisions for complex problems. An example is in how governments responded to COVID-19. For example, '**what if** the Australian government did not initially restrict international travel, what would the COVID-19 infection rates look like?' **In this way, we are hoping to develop a systems model 'what if' tool for mental health so that all young people receive the right care, regardless of who they are or where they are from.** This survey focuses on learning more about your thoughts on what you hope to achieve through your involvement in our research, as well as understanding your opinion on what changes need to be made to improve mental health care for young people in your community. Your response will be completely confidential.*

To learn more about our research, watch the video below:
[Location of online video]
Before continuing, please make sure that you have read the Participant Information Sheet.

a) *Have you read through the Participant Information Sheet?*
   - Yes
     - If *'Yes'* selected: Do you consent to completing this survey?
   - No
     - If *'No'* selected: Please read the Participant Information Sheet.

A. Personal details

1. What is your current age?
   - 14-17 years old
   - 18-24 years old
   - 25-34 years old
   - 35-44 years old
   - 45-54 years old
   - 55-64 years old
   - 65 years old or older
2. Do you identify as any of the following? *Please select all that apply:*
   - Aboriginal and/or Torres Strait Islander
   - Culturally and Linguistically Diverse background
   - Lesbian, gay, bisexual, transgender, gender diverse, intersex and queer (LGBTIQ+)
   - Religious and/or spiritual
   - None of the above
   - Other: Click or tap here to enter text.
3. Which of the following best describes you? *Please select all that apply:*
   - I experienced (or currently experiencing) mental ill-health
   - I help look after a young person to support their mental health (including family member, friend)
   - Aboriginal and Torres Strait Islander Elder
   - Community support (including religious support, cultural support)

Educator (including teacher, school counsellor, principal)
Front-line health services professional (including clinician, practice manager, Aboriginal Liaison Officer)
Health organisation administrator (including staff of Primary Health Network)
None of the above
Other: Click or tap here to enter text.

B. Expectations for the research Program

4. What do you hope to get out of participating in the co-design workshops? *Please select all that apply*:
   Opportunity for my voice to be heard
   Be involved to improve youth mental health care in my community
   Opportunity to be a mental health advocate to support young people in my community
   Better understand youth mental health care available in my community
   Engage with people from other health services/organisations
   Gain understanding about the process of how a 'what if' tool is built using systems modelling to improve mental health
   Opportunities for further collaboration with other health services/organisations
   I have no expectations for the co-design workshops
   Other: Click or tap here to enter text.
5. Have you ever been involved in co-design systems modelling workshops to build a 'what if' tool? *(For our research, we are inviting you to three (3) co-design workshops to build a 'what-if' tool to support better decision making, so that all young people will receive the right care, regardless of who they are or where they are from).*
   Yes
   No
   I'm not sure
6. Have you ever used a systems model 'what if' tool before?
   Yes
   No
   I'm not sure
7. What do you hope will be the benefits of the systems model 'what if' tool for [*name of participating site*]? *Please select all that apply*:
   Improve the health and wellbeing of young people in my community
   Assist to make better decisions to improve youth mental health treatments/programs
   Address the current gaps of youth mental health care in my community
   - *If this option is ticked*: What do you think are the current gaps that exist in your community's youth mental health system (e.g. cannot get an appointment because of long wait lists)? Click or tap here to enter text.

   Improve my understanding of youth mental health care in my community
   Better navigate the mental health services/organisations in my community (e.g. to find the right care, to make correct referrals to other services, etc)
   I have no expectations for the systems model 'what if' tool
   Other: Click or tap here to enter text.

For HREC: The following questions in Sections C, D and E will be delivered in an interactive way, utilising gamification tools, for a more interactive user experience (e.g., use of avatars, progress bar, etc). The included visual diagrams are only a draft and are NOT final. The final visual diagrams will be launched using an online platform, Cogniss (please refer to the research study protocol, 'SLHD_BHP Study Protocol_v4_Jan 2022'). The order of Sections may change, or be shuffled randomly when administered to participants.
Section C is intended for participants who have identified to be working in a professional capacity with young people and Sections D and E are intended for all participants.

C. Your current connections with your community's youth mental health system

(Section C is only for participants who have identified to be working in a professional capacity with young people)

8. The following question aims to understand how you interact with other organisations and/or individuals in your community. This will help us understand how your relationships with others may support youth mental health.

What is the name of the organisation where you work?

Click or tap here to enter text.

9. ***Optional:*** Choose your character. If you want to skip, press 'Continue.'

10. Please select all organisations you have had contact with professionally (current or previous) in the past two (2) years. *You may have contacted these organisation(s) to refer a young person for mental health support, get advice or information, collaborate on a specific task, and etcetera.*
    - ☐ Names of AMS
    - ☐ Names of non-health Aboriginal organisations (land councils, etc)
    - ☐ Names of local schools
    - ☐ Names of local hospitals
    - ☐ Names of local primary care services (including headspace, GP clinics etc)
    - ☐ Names of funding agencies (PHN, ACT Health, etc)
    - ☐ Names of vocational/higher education institutions
    - ☐ Names of special interest groups (LGBTI+, refugee, etc)
    - ☐ Names of police/emergency services
    - ☐ Names of government (city council, etc)
    - ☐ Names of insurers/private service providers

11. ***Optional:*** Are there additional key people and/or organisations that you would like to share that you are working/have worked with professionally in the past two years (current or previous)?
For example, an Indigenous Elder, your local high school, etc. Please exclude people from within your own organisation.

Click or tap here to enter text.

12. *Based on responses identified in Qs 10&11:*
Drag and drop – How often have you spoken to these organisation(s) and/or people?

Daily	Weekly	Monthly	Quarterly	Six-monthly	Yearly

13. *Drag and drop* – How would you describe the nature or type of relationship you have with these organisation(s) and/or people? *You can choose the same organisation and/or person into more than one option.*

**Service provision** *(to refer or receive a service)*	**Advisory** *(to give or seek advice)*	**Working together** *(for example, on a project)*	**Administrative** *(for example managing contracts)*	**Sharing resources** *(for example, information)*	**Other** *(please specify)*

The following question aims to understand how you interact with other organisations and/or individuals in your community.
This will help us understand how your relationships with others may support youth mental health.

What is the name of the organisation where you work?

Test

- optional
Optional: Choose a character to complete the activity.

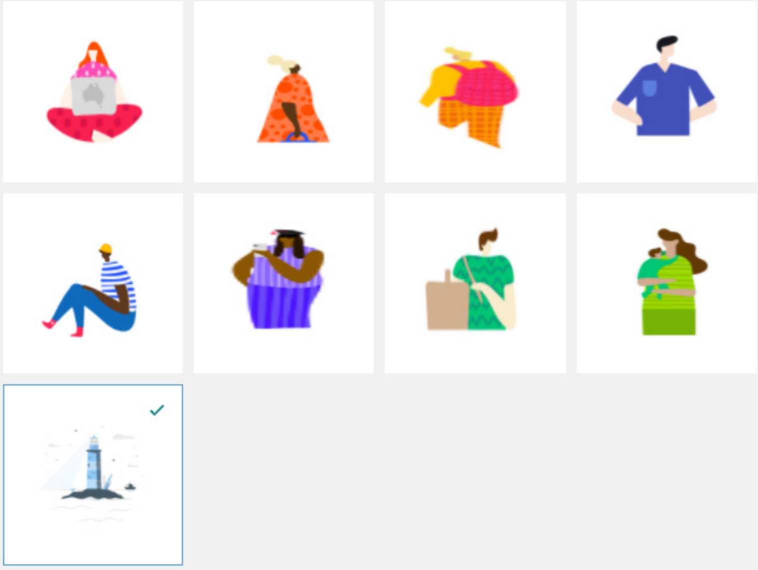

Continue

Please select all organisations you have had contact with (current or previous) in the past two (2) years. *You may have contacted these organisation(s) to refer a young person for mental health support, get advice or information, collaborate on a specific task, and etcetera.*

- Names of AMS ✓
- Names of non-health aboriginal organisation (land councils, etc) ✓
- Names of local schools ✓
- Names of local hospitals ✓
- Names of local primary care services (including headspace, GP clinics, etc) ✓
- Names of funding agencies (PHN, ACT health, etc) ✓
- Names of vocational/higher education institutions ✓
- Names of special interest groups (LGBTI+, refugee, etc) ✓
- Names of police/emergency services ✓
- Names of government (city council, etc) ✓
- Names of insurers/private service providers ✓

- optional

Optional: Are there additional key people and/or organisations that you would like to share that you are working/have worked with in the past two years (current or previous)?

For example, an Indigenous Elder, your local high school, etc. Please exclude people from within your own organisation.

Test

Add another

Continue

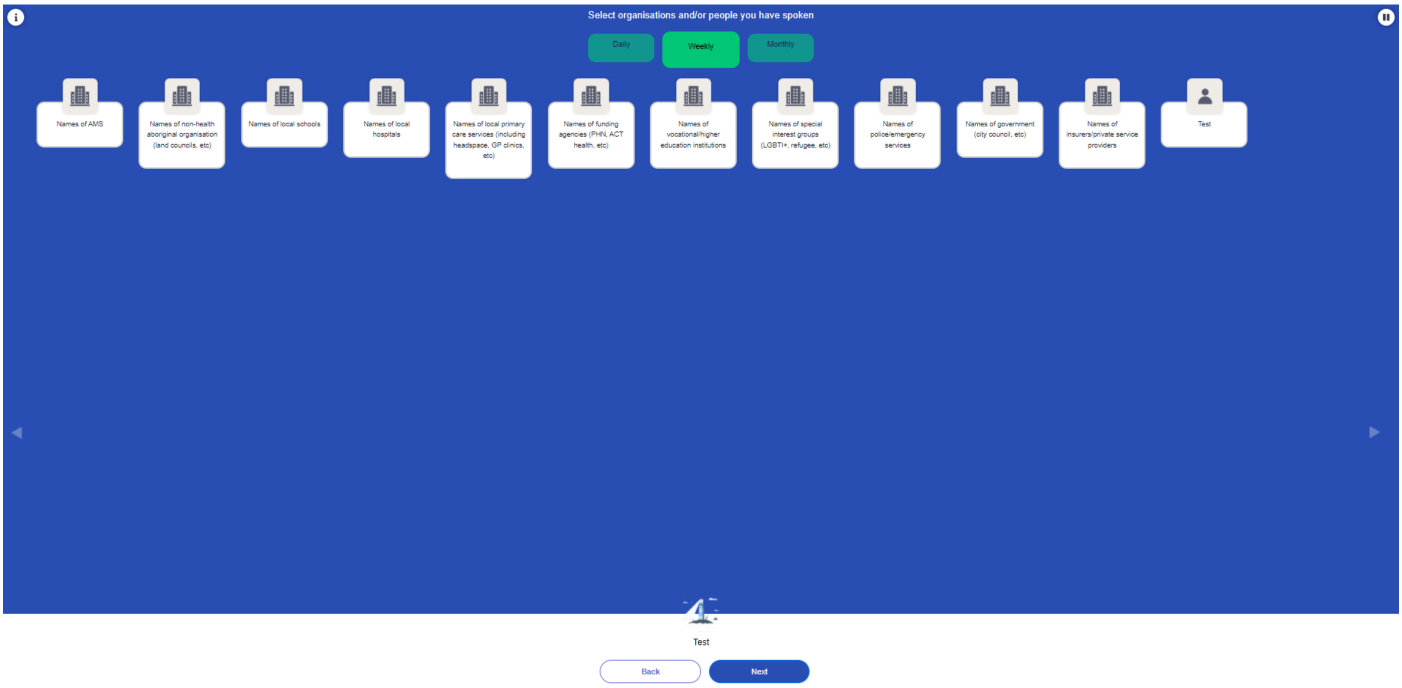

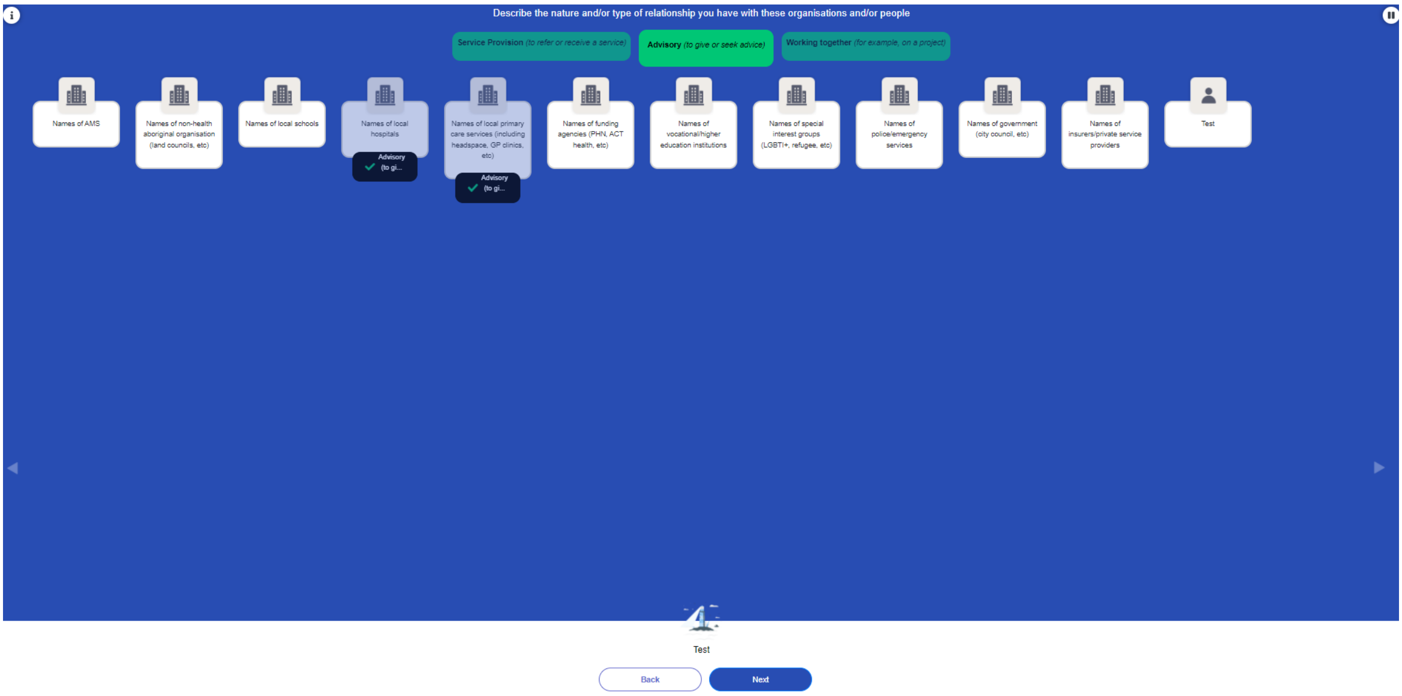

D. Navigating your youth mental health system

For HREC: The case studies may differ slightly to fit the context of participating site. The case studies will be delivered in an interactive way, utilising gamification tools, for a more interactive user experience. Visual diagrams will be launched using an online platform, Cogniss.

Participants may have the flexibility to answer any of questions 13(a-c).

14. The following question aims to understand the experiences of young people accessing mental health services and/or organisations in the *[name of other location]*.
    Please read one of the following case studies and put yourself in the shoes of Annie or KT to help us understand how they should seek help accessing mental health services and/or organisations in your community. Alternatively, you can choose to share your own help-seeking experiences.

    Which story would you like to explore?
    Case study (Annie or KT)

- *If this option is ticked*: Whose story would you like to explore?
  - Annie, 15-year old
  - Kevin Thomas (KT), 23-year old
- A personal experience (mine or someone I care for)

a. **Annie is a 15-year-old** who lives with her mum in *[name of participating site]*. They have recently moved from *[name of other location]* two months after her parents separated. Annie does not talk to her father any more as “he’s very angry” and she feels unsafe with him. Annie is in high school, but struggles to complete work, ‘freezing’ during tests and not handing in homework. She has not made any new friends since moving to *[name of participating site]*, and lost all touch with all her old friends following the move. She hates going on her Instagram and TikTok as all she sees are updates from her old friends having a good time without her. Annie does not feel connected to anyone, and is hoping for help to feel better.

   Based on your knowledge and experiences of the mental health services and/or organisations in the *[name of participating site],* what might a ‘typical’ journey for Annie look like? Please help us continue Annie’s story on how she could seek help.

b. **Kevin Thomas ‘KT’ is a 23-year-old** and has been living in a share house in *[name of participating site]* with no mental health services and limited transport. KT was previously living with his dad before he passed away. KT has a strong connection to his Aboriginal culture through his community. While he feels ‘stuck’ and ‘bored’, he would find it difficult to leave. KT doesn’t speak with his mother and his closest social connections are his housemates, who routinely use ‘ice’ (methamphetamines). KT does some work on the local farm, but he has not been feeling well enough to go to work for two weeks.

   Based on your knowledge and experiences of the mental health services and/or organisations in the *[name of participating site]*, what might a ‘typical’ journey for KT look like? Please help us continue KT’s story on how he could seek help.

c. **Thanks for sharing your personal experience.** We acknowledge that mental health experiences are ongoing and challenging (with lots of ups, downs, twists, and turns). Please share with us about one of your mental health experience on how you accessed mental health services and/or organisations in *[name of participating site]* to seek help.

   Note: We are not asking you to share your entire mental health story with us. Rather, we are hoping to understand one of your mental health experiences you would like to share – it can be the most recent experience, or an experience that has impacted you the most. What actions did you take when you (or your loved one) decided to seek help – what worked and didn’t work?

15. *[Follow-up questions to Q14]:* Which of the following do you think Annie/KT should access // have you accessed in your community when seeking help for mental health support? *Please select all that apply*.

Support	Have accessed? (Tick all that apply)	Can you specify the type of support you received (e.g., Emergency Department, School Counsellor, Sibling, etc)?
**Hospital** (such as Emergency Department)		
**School** (such as at high school, TAFE or university)		
**Walk-in centre** (such as Youth Centres, General Practitioner)		
**Online app help** (such as Calm)		
**Online search** (such as Google)		
**Phone help line** (such as Lifeline)		

**Family and/or friend**		
**Psychology clinic** (such as headspace)		
**Specialist care** (such as from a psychiatrist)		
**Indigenous Health Service**		
**Peer groups** (such as sports team)		
**Religious organisation** (such as a mosque, synagogue, church)		
**Police or other emergency services** (such as calling 000)		
**Don't receive any help** (such as turning to distractions or other coping mechanisms)		
**Other** (please describe who this was)		
I do not wish to access any of the services		

16. *[Follow-up questions to Q15]:* What do you think are some of the challenges young people face to seek help from *[name of support selected]* in your community? *Please select all that apply.*

    Location (e.g. mental health support is located in an area where young person can run into someone they know)
    Opening hours
    Transportation (e.g. no car, no public transport)
    Wait times
    Cost
    Distance from home
    Unable to get referral from a general practitioner (GP)
    Meeting service criteria to make an appointment
    Fear of someone finding out about mental ill-health status
    Help not meeting young people's needs (e.g. cultural safety, not being heard)
    Lack of good quality mental health services/organisations in the community
    Unsure who to trust in the community
    Unsure who to contact in the community
    Young person not feeling worthy of accessing mental health services/organisations
    Young person choosing not to receive care (e.g. they are getting better, avoidance)
    Young person too unwell to get help
    Other: Click or tap here to enter text.

17. Thinking about your own experience, do you think young people in your community have to wait a long time to get into the appropriate mental health service and/or organisation?

    Yes
    No
    Unsure

18. *If 'yes' or 'unsure' for Q17:* What do you think is the average waiting time (e.g. 1 week wait)? Click or tap here to enter text.

19. *If 'yes' or 'unsure' for Q17:* Why do you think young people need to wait a long time to get the appropriate mental health support? Click or tap here to enter text.

20. Thinking about your own experiences, are there times when it seems like you <u>do not</u> have the appropriate mental health services and/or organisations you need in your community?

Yes, we need other services/organisations too
No, we have all the services/organisations we need

21. *If 'yes' for Q20:* What mental health service(s) and/or organisation(s) do you think your community needs? Click or tap here to enter text.

22. How easy do you think that it is for young people to access the right mental health care?

Very difficult to access	1	2	3	4	5	Very easy to access

23. How do you think young people find the right mental health service and/or organisation (e.g. suggestions from family and/or friends, referrals from general practitioner)? Click or tap here to enter text.

E. Hypothetical new youth mental health system for [*name of participating site*]

For HREC: The Program types may differ slightly to best meet the context of participating site.

24. Imagine you had the power to make decisions to improve mental health care in *[name of participating site]*. How would you rate the importance of the program categories below?

**Program type [*programs may be modified based on participating site]***	**How important is this program?**<br>*Drop down:*<br>- *Not important at all*<br>- *Not important*<br>- *Neutral*<br>- *Important*<br>- *Very important*
**Prevention programs focusing on health and wellbeing** (for example, education programs).	
**Early mental health intervention programs** (for example, school counselling services)	
**Community-based mental health intervention programs** (for example, alcohol and/or other drugs support programs)	
**Hospital-based mental health intervention programs** (for example, hospital to home services)	
**Cultural programs** (for example, Aboriginal Community Controlled Health Organisations)	
**Access programs** (for example, creating opportunities to receive better access to care such as afterhours care, or shortened waiting times)	
**Practical employment or educational skills programs** (for example, employment support, financial counselling)	
**Other interventions (Please list):**	

a) *For options where respondent puts down 'not important at all'; 'not important';* ***or*** *'neutral':* Can you please explain? Click or tap here to enter text.

b) *For options where respondent puts down 'important'* ***or*** *'very important':* Please rate the importance of the following individual programs:

**Program type [*programs may be modified based on participating site]***	**How important is this program?**<br>*Drop down:*<br>- *Not important at all*<br>- *Not important*<br>- *Neutral*<br>- *Important*<br>- *Very important*
**Prevention programs focusing on health and wellbeing**	
School-based programs led by young people focused on mental health and wellbeing education	
Community campaigns to decrease stigma, increase knowledge, and promote willingness to seek help	
Suicide prevention specific to local community	
Programs to increase social connections and reduce isolation	
Engagement of young people with lived experience in all aspects, including the development and implementation of suicide prevention plans	
Activities to reduce the marketing and consumption of alcohol and/or other drugs	
Education programs to increase the number of trained mental health professionals in the community, including people with lived experience (such as peer workers)	
Aboriginal prioritised and led prevention programs for Aboriginal communities	
Other (please specify): Click or tap here to enter text.	
**Early mental health interventions programs:**	
Online support targeting young people (e.g. Reachout and Beyond Blue chat lines)	
Primary health care (e.g. care provided by General Practitioners)	
School counselling services	
Other (please specify): Click or tap here to enter text.	
**Community-based mental health intervention programs**	
Face-to-face counselling support for young people (e.g. headspace)	

Family support programs and counselling	
Suicide helpline and callback service (e.g. Kids Helpline)	
Aboriginal prioritised and led mental health intervention programs for Aboriginal communities	
Alcohol and/or other drugs support programs	
Outreach services following a suicide attempt	
Other (please specify): Click or tap here to enter text.	
**Hospital-based mental health intervention programs**	
Community mental health services to provide a safe space alternative for young people presenting to Emergency Departments (e.g. Safehaven café, Crisis Now)	
Hospital-based mental health services	
Hospital to home services	
Emergency Departments	
Other (please specify): Click or tap here to enter text.	
**Cultural programs**	
Culturally appropriate services, such as Aboriginal Community Controlled Health Organisation	
Aboriginal prioritised and led interventions for the Indigenous community	
Training the whole mental health workforce on cultural safety	
Other (please specify): Click or tap here to enter text.	
**Access programs**	
Increased access to specialist psychiatry consultations	
No barrier primary health services (immediate access/no referral/no cost), such as a drop-in service	
Reduced waiting time for specialist care (such as psychiatrists)	
After hours mental health in-home community based services	
After hours services for people who are busy during the day with work or school	
Digital technologies to connect young people to mental health professionals	
Online apps to self-manage mental health	
Directory of all the mental health services in the local community	
Mental health services to be better connected to support referral journey for patients	
Other (please specify): Click or tap here to enter text.	
**Practical employment or educational skills programs**	
Community youth programs focused on building skills (e.g. employment assistance programs, education support programs)	
Training for community members on suicide prevention (such as for hairdressers, Elders, Ministers)	
Mental health training for all frontline staff (such as General Practitioners, police, paramedics)	
Education courses about mental health and recovery to increase young people's knowledge and skills to make them feel more confident to manage their mental health and wellbeing	
Financial counselling	
Other (please specify): Click or tap here to enter text.	

25. How confident do you feel that the programs you chose as '*important'* **or** *'very important'* will be effective in improving the mental health outcomes of young people in your community?

Not at all confident	1	2	3	4	5	Very confident

F. Experience completing the survey

26. How would describe your experience completing the survey? *Please select all that apply.*

The interactive activities made it more enjoyable to complete the survey
The interactive activities made it more confusing to complete the survey
The survey was too long
The survey was no different to any other survey I have completed previously
Other: Click or tap here to enter text.

Thank you for completing the survey! Which $20 voucher would you like to receive?
Woolworths
BIG W
Caltex
Coles
Target
Kmart
JB Hi-Fi
Prepaid Mobile Recharge
