# Supplementary material for: Participatory Systems Modelling for Youth Mental Health: An Evaluation Study Applying a Comprehensive Multi-Scale Framework
Source: Int J Environ Res Public Health. 2022 Mar 28;19(7):4015. doi: 10.3390/ijerph19074015 (PMC8998357; doi:10.3390/ijerph19074015)
Supplement: Supplementary file 1 [file ijerph-19-04015-s001.zip › Lee GY_PSM evaluation plan YMH Program_SUPPLEMENTARY DOC 3.pdf]

SEMI-STRUCTURED INTERVIEW QUESTIONS TO OBTAIN KEY INFORMANT VIEWS

BASELINE *(PRE-WORKSHOP 1*)

Thank you for agreeing to participate in this research Program and for taking the time to have a chat with me. You have been invited to participate in this research Program because we believe that your experiences are important, and we want your voice to be heard. As part of this research Program, you are invited to three co-design workshops where you will interact with many different people from various backgrounds to discuss the challenges and solutions for youth mental health care. Co-design means that you will be considered an equal to everyone in the workshops – your experiences and opinions will be listened to and everyone will be treated with respect.

*This interview will take up to one hour. To make sure that I accurately reflect your response, I was wondering if you would be okay with this chat being recorded? This recording will only be used for my own record keeping. Also, no one will be able to link your answers back to you, as our research team will never report on individual answers. (*Interviewer to also to go through full informed consent process, including Consent Form*)*

*Just to provide you with some background information, this research Program explores the application of systems modelling to develop a '**what if' tool**, through a co-design process, to improve youth mental health care. Systems modelling allows people to understand **'what if'** scenarios to help make better decisions for complex problems. An example is in how governments responded to COVID-19. For example, '**what if** the Australian government did not initially restrict international travel, what would the COVID-19 infection rates look like?' In this way, we are hoping to develop a systems model 'what if' tool for mental health so that all young people receive the right care, regardless of who they are or where they are from.*

In today's interview, we are interested to learn more about your thoughts on what you hope to achieve through your involvement in our research Program, as well as to understand your opinion on what changes need to be made to improve mental health care for young people – e.g. easier to find – in the [name of participating site] region.

<u>Current youth mental health care</u>

1. Based on your experience, what are the current challenges of youth mental health care in [name of participating site]?
2. What do you think are driving these challenges?
3. What changes do you think are required to improve youth mental health care?
4. Why do you think these changes have not yet been implemented?
5. Which prevention or treatment programs would you prioritise to manage youth mental health in your community? Why?

Decision-making: *The next questions focus on your opinions about how decisions are made that affect youth mental health care in your community.*

6. ***FOR PROFESSIONALS ONLY:***
    - Can you tell me about how you or your organisation makes decisions to provide youth mental health support in your community?
    - What sort of evidence is used to inform these decisions?
7. ***FOR YOUNG PEOPLE AND CARERS ONLY:***
    - How do you think decisions are made to provide youth mental health support in your community?
    - How do you think decision making for youth mental health in your community can be improved? What is working well?
8. Are there certain groups or people who are very influential in decision making specific to youth mental health in your community? Can you tell me who they are and why they are so influential? How do you think they make their decisions? Do you think they use evidence? How do you think this impacts mental health care in your community?
9. Are there groups or people who are “missing” from decision processes whose voices should be heard more?
    - *[If reply ‘yes’]:*
        - Can you please indicate who they are and why their voices are not considered? Why is it important to listen to them when making decisions that affect youth mental health care in your community?

Previous experiences & Expectations

10. What previous experience do you have with systems modelling (with or without co-design)?
    - *[If reply ‘yes’]:*
        - Could you tell me about the systems modelling process and your experience of it?
        - In your opinion, what are the benefits and limitations of systems models as a ‘what if’ tool?
        - *[If developed through co-design]:* What were some of the benefits and challenges from working with others during the workshops?
        - *[If not developed through co-design]:* How do you think a co-design process through workshops would have impacted the development of the systems model?
        - What are you hoping to get out of participating in this research Program? What value do you think this research Program will have?
        - How much do you think the systems model ‘what if’ tool will improve and/or support decision-making for youth mental health care in your community? How?

- What are you hoping to get out of participating in this research Program? What value do you think this research Program will have? What is needed to make sure that your expectations are met?

- *[If reply 'no']:*
  - What are you hoping to get out of participating in this research Program? What value do you think this research Program will have? What is needed to make sure that your expectations are met?
  - What do you think are some benefits you expect from working with others during the workshops?
  - What do you think are some challenges you expect from working with others during the workshops?
  - How much do you think the systems model 'what if' tool will improve and/or support decision-making for the mental health services and/or organisations in your community? How?

Current connections

11. ***FOR PROFESSIONALS ONLY:***
    - Which organisations or people in your community's youth mental health system do you interact with? How do you interact with them?
    - How important are these relationships for your organisation and/or your work?
    - How do you think the co-design systems modelling workshops may impact these relationships?
    - What do you think your community is doing well in terms of working with each other to improve youth mental health?
    - What do you think are the biggest challenges in terms of working with each other to improve youth mental health?

12. ***FOR YOUNG PEOPLE AND CARERS ONLY:***
    - How do young people seek help in your community?
    - How do young people navigate your community's mental health services (e.g. is it easy to navigate, is it quick to get into an appointment)?
    - What do you think your community is doing well in terms of offering youth mental health support?
    - What do you think are some of the barriers for young people to access mental health services in your community?

Recommendations

13. Do you have any other comments or questions regarding this research Program?
