# Supplementary material for: Participatory Systems Modelling for Youth Mental Health: An Evaluation Study Applying a Comprehensive Multi-Scale Framework
Source: Int J Environ Res Public Health. 2022 Mar 28;19(7):4015. doi: 10.3390/ijerph19074015 (PMC8998357; doi:10.3390/ijerph19074015)
Supplement: Supplementary file 1 [file ijerph-19-04015-s001.zip › Lee GY_PSM evaluation plan YMH Program_SUPPLEMENTARY DOC 4.pdf]

SEMI-STRUCTURED INTERVIEW QUESTIONS TO OBTAIN KEY INFORMANT VIEWS

FOLLOW-UP (*POST-WORKSHOP 3 <u>AND</u> 6-MONTHS POST-WORKSHOP 3*)

*Thank you for being involved in the co-design workshops – your participation has been invaluable. Thank you, also, for taking the time to have a chat with me today. This interview will take up to one hour. To make sure that I accurately reflect your response, I was wondering if you would be okay with this chat being recorded? This recording will only be used for my own record keeping. Also, no one will be able to link your answers back to you, as our research team will never report on individual answers. (*Interviewer to also to go through full informed consent process, including Consent Form*)*

*To refresh your memory, this research Program aims to improve mental health care for young people (e.g. easier to find) in the [name of participating site] region. During the co-design workshops, we are working/have worked together to develop a **'what if' tool** through the application of systems modelling. Systems modelling allows people to understand **'what if'** scenarios to help make better decisions for complex problems. An example is in how governments responded to COVID-19. For example, '**what if** the Australian government did not initially restrict international travel, what would the COVID-19 infection rates look like?' In this way, we are hoping to develop a 'what if' tool for mental health so that all young people receive the right care, regardless of who they are or where they are from in your community. The interview today will focus on your thoughts on how you found the process of developing the systems model 'what if' tool, how we can improve in the future, and what your observations have been about youth mental health care in your community.*

<u>Experience participating in this research Program</u>

1. How was your experience participating in the co-design workshops to build the systems model 'what if' tool? What did you hope to get out of participating in the co-design workshops? Have your expectations been met by participating in this research Program?
2. Do you think that workshop participants reflected the diverse mental health stakeholders that represent your community's mental health system? Why or why not?
3. How many workshops did you attend?
    - *[If less than 3]*: Was there a reason why you could not attend all three workshops?
4. What do you think were the strengths of the co-design process to build the systems model 'what if' tool?
5. What can we do to improve?
6. Based on your experience, would you say the co-design process to build the systems model 'what if' tool was worthwhile of your time? Did you learn something that will/already has benefited you?
7. Do you think you will participate in other similar research Programs in the future? Why or why not?

<u>Experience using the systems model 'what if' tool</u>

8. *The following questions will ask about your experience with the systems model 'what if' tool:*

- Does the systems model 'what if' tool represent youth mental health care for your community? Why or why not?
- Does the systems model 'what if' tool generate useful insights? Why or why not?
- Do you think there was transparency around how the systems model 'what if' tool was built? Why or why not?

9. Are you confident in using the systems model 'what if' tool (e.g. it is easy to use)?
    - *[If reply 'yes']:*
        - What has helped you to understand how to use the systems model 'what if' tool?
        - In your opinion, what would you say are facilitators and barriers to the use of the systems model 'what if' tool?
        - Do you have any hesitations when it comes to using the systems model 'what if' tool? *(if yes: can you tell us a little more about this?)*
    - *[If reply 'no']:*
        - What could have been done to help you better understand how to use the systems model 'what if' tool?
        - Did you want to become competent users of the systems model 'what if' tool? Why or why not?
        - In your opinion, what would you say are facilitators and barriers to the use of the systems model 'what if' tool?
10. To your knowledge, have the outcomes of the systems model 'what if' tool been used to improve youth mental health care in your community (e.g. support applications for funding/investment cases; advocate for greater investments to support youth mental health, and/or; build community support for economic, social, educational or mental health programs)?
    - *[If reply 'yes']:*
        - Can you give us some examples?
        - How do you think the systems model 'what if' tool supports this process? (*prompt: were there any benefits and challenges?*)
    - *[If reply 'no']:*
        - Do you think there are outside factors that have influenced the extent to which the systems model 'what if' tool has been used to improve youth mental health care in your community?
        - Do you think the systems model 'what if' tool can add valuable insights to future applications in your community? Why or why not?
11. Based on your experience, would you say systems modelling is worthwhile for mental health communities in other regions? Why or why not?
12. Have you experienced a shift in your thinking about youth mental health care in your community (and its associated services/organisations) as a result of the systems modelling process?
    - *[If reply 'yes']:*
        - What has changed in your thinking? (*prompt: where there might be gaps in services?; how to best use resources to prevent and treat youth mental health?*)
        - How have/will these changes impact the decisions you make?
    - *[If reply 'no']:*
        - Did you benefit from interacting with others during the workshops?

Current connections

13. How was it working with others during the co-design systems modelling workshops?
14. What were some of the benefits from working with others during the workshops?
15. What were some of the challenges working with others during the workshops?
16. ***FOR PROFESSIONALS ONLY:***
    - Which organisations or people in your community's youth mental health system do you interact with? How do you interact with them?
    - How important are these relationships for your organisation and/or your work?
    - How do you think the co-design systems modelling workshops may impact these relationships?
    - What do you think your community is doing well in terms of working with each other to improve youth mental health?
    - What do you think are the biggest challenges in terms of working with each other to improve youth mental health?

17. ***FOR YOUNG PEOPLE AND CARERS ONLY:***
    - How do young people seek help in your community?
    - How do young people navigate your community's mental health services (e.g. is it easy to navigate, is it quick to get into an appointment)?
    - What do you think your community is doing well in terms of offering youth mental health support?
    - What do you think are some of the barriers for young people to access mental health services in your community?

Recommendations

18. Do you have any other recommendations or comments regarding this research Program (e.g. recommendations for future systems modelling work, suggestions to improve the 'what if' tool development process)?
